# Involving citizens in priority setting for public health research: Implementation in infection research

**DOI:** 10.1111/hex.12604

**Published:** 2017-07-21

**Authors:** Timothy M. Rawson, Enrique Castro‐Sánchez, Esmita Charani, Fran Husson, Luke S. P. Moore, Alison H. Holmes, Raheelah Ahmad

**Affiliations:** ^1^ National Institute for Health Research Health Protection Research Unit in Healthcare Associated Infections and Antimicrobial Resistance Imperial College London Hammersmith Campus London UK; ^2^ Health Group Management Department Imperial College Business School London UK

**Keywords:** infection funding, patient & public engagement, strategic decision making

## Abstract

**Background:**

Public sources fund the majority of UK infection research, but citizens currently have no formal role in resource allocation. To explore the feasibility and willingness of citizens to engage in strategic decision making, we developed and tested a practical tool to capture public priorities for research.

**Method:**

A scenario including six infection themes for funding was developed to assess citizen priorities for research funding. This was tested over two days at a university public festival. Votes were cast anonymously along with rationale for selection. The scenario was then implemented during a three‐hour focus group exploring views on engagement in strategic decisions and in‐depth evaluation of the tool.

**Results:**

188/491(38%) prioritized funding research into drug‐resistant infections followed by emerging infections(18%). Results were similar between both days. Focus groups contained a total of 20 citizens with an equal gender split, range of ethnicities and ages ranging from 18 to >70 years. The tool was perceived as clear with participants able to make informed comparisons. Rationale for funding choices provided by voters and focus group participants are grouped into three major themes: (i) Information processing; (ii) Knowledge of the problem; (iii) Responsibility; and a unique theme within the focus groups (iv) The potential role of citizens in decision making. Divergent perceptions of relevance and confidence of “non‐experts” as decision makers were expressed.

**Conclusion:**

Voting scenarios can be used to collect, en‐masse, citizens' choices and rationale for research priorities. Ensuring adequate levels of citizen information and confidence is important to allow deployment in other formats.

## 1 INTRODUCTION

Between 1997 and 2010 £1.4 billion of public funds were invested in infection research in the United Kingdom (UK)^1^ comprising the major source of funding (54% of the overall funding). Despite the majority of funding coming from public sources, UK citizens currently have no formal role in determining where or how this money is invested within this field of research.

The Health Research Authority (HRA) in the UK has set out clear aims and objectives for involving patients and the public in the research process, setting out a clear mandate for improving patient and public involvement (PPI) directly in research.^2^ Within the field of infection, research organizations and funders have engaged widely with PPI at the levels of “direct care” and “organizational, design and governance”.^3^ However, PPI is less present in “policy” aspects of infection research as described by the framework for patient and family engagement in health and health care proposed by Carman and colleagues.^3^ There is also a lack of tested approaches for setting research priorities for use by public agencies who make funding decisions.^3^ In this sense, PPI at the policy level seems to stop at informing patients and the public about research but does not partner them in the decision process of setting priorities for research, and hence a long way from citizen‐led priority setting.^4^ Despite a growing body of literature exploring citizen‐led priority setting,^5^, ^6^, ^7^, ^8^, ^9^ there is little evidence to support the feasibility of large‐scale interventions to collect views of citizen priorities for infection research. Greater understanding of how to facilitate such an intervention and the potential challenges and biases associated with this is yet to be defined.

To test the feasibility of partnering citizens in priority setting, there first of all needs to be a clear and validated method for informing and involving a large number of citizens in the decision process. This must take into account many of the common issues associated with researcher engagement with patients and the public and sharing decision making across research and health care.^10^, ^11^, ^12^, ^13^ An important contextual factor for any PPI activity is the health literacy of the population; in the UK for example, approximately 43% of citizens require assistance in understanding written health information.^10^, ^14^, ^15^ This means that as well as ensuring that issues of access and participation are addressed on a logistical level, involvement at the strategic level runs the risk of tokenistic or technocratic involvement, if methods are not fit for purpose.^16^, ^17^, ^18^ Citizens must have time and space to express their information needs and levels of confidence when asked to participate in decision‐making processes.

We set out to develop a tool and test its feasibility as part of a reproducible methodology capable of exploring citizen priorities for infection research from a large population sample, with the potential to inform priorities across research programmes.

## 2 METHOD

### 2.1 Scenario design

A case scenario (Appendix S1) was created as a means of accessing a large, sample of citizens' views on their priorities for research funding in the field of infection. The scenario was created in consultation with a number of researchers with experience in PPI and health literacy, acting as a working group for this project (TMR, EC, ECS, LSPM, AH and RA). The scenario was then critiqued by the unit's patient representative who has experience of developing decision support tools and evaluating patient facing interventions. She provided structured feedback on the dimensions of internal validity, consistency of presented information, and assuring bias was not introduced in the way that the working group presented the information. Following revisions, the scenario was then piloted on two non‐medical support staff within the research department, a junior doctor, and five citizens through social networks not involved in health‐care delivery or research. The aims of the scenario were to put citizens visiting a university open day event in the position of a large global charity, with the means to allocate funding to one or two research areas within the theme of infection. During this scenario, the citizens would be presented with information on six infection research areas or “finalists” from within this theme and would be given the opportunity to allocate funding to one or two of six research areas. Funding was selected at 2x £50 million (equal to 1 vote each) or 1x £100 million (equal to 2 votes) allocated to two or one of the finalists, respectively. Citizens were also required to write a brief justification for funding allocation decisions on the back of each cheque that they wrote.

After development of the scenario, the working group developed a poster to implement the scenario at the university public festival. The six “finalists” in the scenario were selected following a review of the literature. The review was aimed at identifying six infection research areas with a variable burden on both global and local health‐care systems. The six selected research areas were as follows; HIV/AIDS, malaria, tuberculosis, drug‐resistant infections, influenza and emerging infections (such as Zika and Ebola). A world map was created with these six finalists positioned across it. A set of key metrics were selected by the working group and included in the poster display. To enable wide participation and comparability of information, the metrics were displayed graphically in a Likert‐like manner. This was guided by the literature, in particular, the recent Wellcome Trust research on drug‐resistant infections, which demonstrated that citizens find large numbers and figures difficult to associate with^19^ as well as qualitative work on how the public engage with graphical metrics.^20^ Four consistent pieces of information were selected for translation into representative Likert 5‐point scales to be used across all six research areas. These were as follows; the number of people affected annually, current number of deaths annually, the burden on the UK health‐care system and the burden on health‐care globally. A key individual point was then selected from the literature for representation on each of the six selected research areas, with the exception of emerging infections, where it was felt that examples of these would be more appropriate (ie Zika virus and Ebola). The remaining five points that were selected were as follows; HIV/AIDS—new cases per year; malaria—new cases per year; tuberculosis—burden of treatment; drug resistance infections—potential deaths per year; influenza—pandemic potential.

### 2.2 Scenario implementation & analysis

The scenario was set up and tested across 2 days on 7th and 8th May 2016 at Imperial College London university public festival, which was visited by over 15 000 members of the public. A stand was set up in one of the festival buildings within the infection zone, which had research presented on all six research areas within our scenario. One researcher (TMR or EC or RA) manned the scenario over a two‐day period, with short vignettes provided to guide what information could be given to individuals who sought further clarification during participation in the scenario.

Following each day of the festival, all cast votes and justifications for why individuals allocated funding were collated and analysed using NVIVO pro11.0 software, to analyse voting habits and the major drivers of voting choices. Voting practices and justifications were compared across both days to assess them for reproducibility.

### 2.3 Focus group evaluation

Twenty citizens (recruited through Cherry Picked, UK—a specialist qualitative recruitment company) were invited to participate in a 1‐hour in‐depth focus group on May 16th. Citizens were recruited using specific screening criteria, from a database of 20 000 individuals from around the UK who had signed up with the recruitment agency previously. The screening criteria, which included information such a demographics and location, as well availability during the days/hours that the workshop was to be held, identified a sample of 500 individuals. An initial email was sent to all 500 individuals, advertising the workshops. The respondents were then stratified according to recruitment criteria, and 20 individuals were selected for inclusion. For this evaluation, we aimed for an equal mix of genders, educational statuses and age groups as well as a diverse mix of ethnicities. Citizens must not have attended the university public festival. Two further emails were sent to these individuals confirming their attendance and sending directions to the venue. The focus groups aimed to explore the participants' views on PPI in research policy decisions, to explore methods for collecting citizen views on research priorities, and evaluate the method and results from our pilot of the scenario at the public festival.

One researcher (ECS) led three separate focus groups, following a topic guide (Appendix S2). A second researcher (TMR) observed the focus groups, making notes on the session to allow areas of researcher reflexivity to be considered and addressed during data analysis. The questions were developed by five researchers highly experienced in qualitative research but from diverse health and social science backgrounds (TMR, ECS, EC, LSPM and RA) and were then pre‐tested with a patient representative with experience of qualitative research (having previously co‐authored with the research team). As with the scenario development, these were then piloted on two non‐medical support staff within the research department, a junior doctor, and five citizens through social networks, not involved in health‐care delivery or research. The questions were open ended and included prompts. In the focus groups, space was provided for individual perceptions and opinions to be aired. There were no normative statements of what the public should or should not do—instead the participants were asked to give their views on whether the public should be involved at all in the decision making, how they should be involved, and if the tool provided was clear and suitable for use with members of the public.

All participants were consented, and discussions were audio recorded and transcribed verbatim. These were then analysed using a mixed inductive and deductive technique, using NVIVO pro11.0 software. For inductive analysis, one researcher (TMR) reviewed all transcripts allowing initial codes to be generated by line by line coding for first order codes, and a second researcher (LSPM) independently coded transcripts.^21^, ^22^ During line by line coding, the comments provided by the observer were considered with the aim of complementing and balancing areas of reflexivity derived from the analysts' own background, beliefs and prior experiences.^23^ After comparing the coded transcripts, a list of emerging categories were developed in addition to the codes generated through analysis of the public festival voting. After meeting and agreeing on key categories and themes within the text, two researchers (TMR & RA) independently preceded to systematically cross‐review the text, coding passages based on these agreed codes and categories, subsequently grouping them into overarching themes. On review, any discrepancies were discussed within the working group, including our patient member (FH), and consensus reached. Examples of key opinions and ideas from the text for each main theme identified were then charted to allow mapping and interpretation of the results.^21^

This project was reviewed by the regional ethics committee, who deemed that citizen interviews during focus groups and festival involvement (anonymous voting and written justifications) did not require ethical approval and was allowed under the local clinical governance and research compliance office oversight. Written consent was obtained from the focus group participants. Participants providing anonymous votes and written justifications at the festival were not required to provide written consent.

## 3 RESULTS

### 3.1 University public festival voting

**Figure 1:**
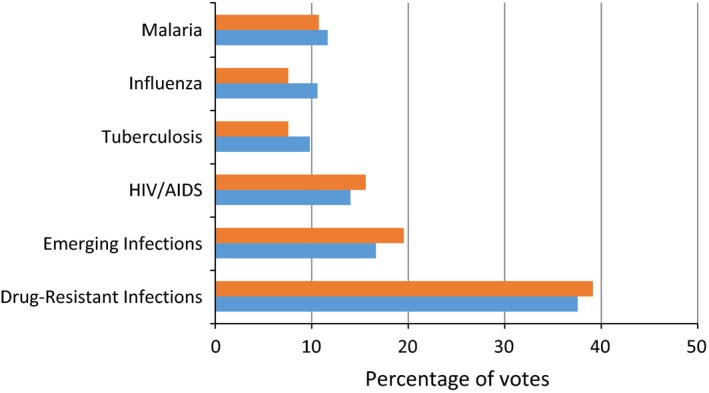
Comparison of the percentage of votes cast by citizens participating in voting for their priorities for infection funding across two days. Orange = day 1; blue = day 2

Over the two‐day period that voting occurred, approximately 3000 citizens visited the building where the tool was deployed. In total, 491 votes were cast by 246 individual citizens, representing the majority of the visitors to the stand. The stand itself was popular with 8% of the total visitors across the 2 day event visiting the stand. Voting was reproducible over both days with drug‐resistant infections receiving the most votes across both days (100/266; 38% day 1 & 88/225; 39% day 2). Emerging infections was second (44/266; 17% day 1 & 44/225; 20% day 2) and HIV/AIDS third (37/266; 14% day 1 & 35/225; 16% day 2) over both days (Figure 1).

Table 1 provides a breakdown of categories and themes that arose from analysis of these justifications for citizen voting habits. Three themes emerged from the categories reported on the voting slips. These were (i) information processing (I), which included the relevance of the infection to the individual, its geographical proximity and also how information has been presented to those individuals; (ii) knowledge of the problem (K), which includes our current understanding of the problem, what solutions/treatments are available, and areas where greater understanding/research is required; and (iii) responsibility (R), which includes the individuals person feeling of responsibility to act, feelings of the need to help others, and responsibility to future citizens and public health in general.

**Table 1:** Common voting categories and themes identified as driving citizens' decisions for individual funding allocations at the university festival

Drug‐Resistant Infections (theme)	Votes	Emerging Infections	Votes	HIV AIDS	Votes
Potential impact on society (**R**)	36	Unknown (**K**)	20	High mortality (**R**)	12
Low funding (**K**)	26	Potential impact globally (**R**)	13	Affects poor countries (**R**)	7
Increasing problem (**I**)	16	Dangerous (**I**)	11	High global burden (**R**)	6
Apocalyptic potential (**I**)	13	Being prepared (**K**)	5	Not enough funding to support poor countries (**R**)	5
High mortality (**I**)	12	Prevention (**I**)	4	High incidence (**K**)	4
Global impact (**R**)	11	Effects the poor (**R**)	3	Need to eliminate (**K**)	3
Prevention (**I**)	4	Urgent need for action (**I**)	2	Can cause other diseases (**R**)	3
Area of personal interest (**K**)	4	Potential to effect the individual (**I**)	2	Personal experiences (**I**)	1
Potential danger to the individual (**I**)	3	High mortality (**R**)	2	Importance to me (**I**)	1
Local impact in UK (**I**)	3	Building capacity to cope (**K**)	1	Fear (**K**)	1
Lack of knowledge on subject (**K**)	3			Effects children (**R**)	1
Avoidability (**K**)	3				
Fear of them (**K**)	1				
As we have created this problem (**R**)	1				
A complex problem (**K**)	1				
Influenza	Votes	Malaria	Votes	Tuberculosis	Votes
Pandemic potential (**I**)	10	Deaths per year (**R**)	8	High mortality (**R**)	13
Lack of funding (**K**)	6	Effects poor countries (**R**)	7	High treatment burden on patients (**K**)	3
Potential impact on society (**R**)	4	High numbers of people infected (**K**)	6	Increasing issue (**K**)	2
High local burden in UK (**I**)	2	Global burden (**R**)	5	Effects the poor (**R**)	2
Prevent pandemics (**R**)	1	Need new treatments (**R**)	4	Prevention (**K**)	1
Poor countries need support (**R**)	1	Low funding (**R**)	4	I know many people affected (**I**)	1
No cure (**K**)	1	I am from a country affected by this problem (**I**)	2	Growing problem of resistance (**R**)	1
Lack of research currently (**K**)	1	Prevention (**K**)	1	Global burden (**R**)	1
Incidence of infections (**K**)	1	Prevent resistance to treatment (**R**)	1	Affects children (**R**)	1
Area of personal interest (**K**)	1	Dangerous (**I**)	1		
		Can be transmitted (**K**)	1		

### 3.2 In‐depth evaluation of the intervention

Of the 20 citizens who participated in the focus groups there was an equal split in gender and ethnicities with five citizens aged 18‐25 years, five 26‐40 years, five 41‐64 years and five aged 65+ years. Each group participated in two 30‐minute focus groups with a short break between both sessions. Participants found the tool clear and were able to make comparisons based on the information within the poster. Points of clarification sought included whether the geographical distribution for the themes was definitive for all six themes and clarification of the “size” described by the figures which had been translated into Likert‐like scales for comparison. Table 2 summarizes themes and categories that emerged during the focus groups. These were similar in nature to the results of the citizen voting held previously, with the exception of a further theme that emerged during the interviews. This was surrounding the citizen's role in decision making for strategic decisions that included categories relating to the individuals willingness to act as a decision maker and their willingness to defer decisions to “experts”.

**Table 2:** Themes and categories from citizen workshops held to explore the citizens' role in setting priorities for research in the theme of infection

Theme	Sub‐categories	Supporting quotes
Information Processing	Relevance to me, the individual Proximity to where I live How information is presented to me	*“It's my money so I have to know what they're doing with it, but saying that I might be a bit selfish because how many times do I go to Asia? I want the treatment to focus on where I live!”*
		*“Something in Europe could easily spread to the UK and affect me”*
		*“Ebola and Zika is not really an issue here [UK]”*
		*“Wouldn't it be more beneficial, though, to go to where you know that there's going to be an effect, where you know where the burden is going to be quite high on the NHS”*
Role in Decision Making	Deferring decisions to experts Willingness to act as a decision maker	*“I wouldn't want to make the decision as part of the general public. I would like to have confidence in who's making the decisions”*
		*“We're the laymen. We can't make decisions”*
		*“They are the experts, I think they should make the decision. We should put our faith in them and if they mess up, well then we know who to blame”*
		*“I would love some transparency, to be able to have some decision making in where the money goes”*
		*“It should be ultimately up to the people whose profession it is, but I do think that the information should be more easily accessible”*
Knowledge of problem	Prior understanding of the problem Curable disease/solutions available Need for greater understanding	*“To not put money into research, to not put funding into something which is unknown and that could potentially kill quite a few people would be a bit naïve, a bit silly of us.”*
		*“The question is do you put money into research for something they haven't found a cure, or money in stopping diseases that have got a cure.”*
		*“We're hearing that it's [tuberculosis] coming back, yeah?”*
		*“You can take a pill that cures malaria, I think. It's not a killing disease nowadays.”*
		*“Likewise HIV, as a bunch of people just pointed out before, there's a, it's a lot about education. There's a lot, there is a lot already out there.”*
Responsibility	Personal feelings of responsibility My ability to help those less fortunate Responsibility to future citizens and the public's health	*“Back in Africa, they know they should be using condoms but they decide not to. So how about put some of the money in certain areas where it's not their fault.”*
		***“**We hear a lot about Ebola and the Zika virus and we've heard not very great things, but I think just because it is in Africa doesn't mean our funding can't go a long way to help them.”*
		*“I think there's also, probably coming from you, some emotion about, oh, look, this is all the West. And here's poor Africa. And that's another emotion thing, do you know what I mean?”*
		*“The biggest death rates are like HIV, which is preventable, and TB, which is obviously in other parts of the world”*

During the interviews, a balance was observed between altruistic feeling of responsibility to help those less fortunate or in greater need vs addressing issues close to home, or that may affect the individual, when making decisions about allocation of funding for research (Table 2, “information processing” and “responsibility themes”). This was further supported by feedback that the location that themes were placed on the world map had influenced some of the decisions that individuals made based on their proximity to Europe, or their perceived threat to selves.

“I think I'd split it between influenza and emerging infections. I feel, with influenza, it's not got much funding, pandemic potential is pretty high, and so something in Europe could easily spread to England and it could affect me. But I also think just emerging infections can be quite, we hear a lot about Ebola and the Zika virus and we've heard not very great things, but I think just because it is in Africa doesn't mean our funding can't go a long way to help them. It is the pandemic potential, the global burden and the local burden, all question marks, but I do feel like we, because we don't know how far it could spread, we don't know its potential, if we put some, if we put a little bit of money into researching those then we could potentially save loads and loads of lives.” [Female participant, 41‐64 years]

The means of displaying information was described as clear and understandable allowing for direct comparison between themes presented in the scenario. However, there were concerns that linked back to the responsibility that lay with the decision maker (individual citizen) in making big choices about where funding should be allocated. Whilst there was agreement that greater transparency and clarity on where money is spent on research was needed, the participants struggled to agree on the overall role that citizens should have in informing final decisions on allocation of resources. Here, over half of the citizens described feeling that the final decisions on allocation of resources should be trusted to those who they saw as “experts” in the field, and thus could weight up all of the information available to make informed decisions about the effectiveness of investment in that area. Other participants disagreed however, and felt citizens ought to have a right to say where research funding, to which they may have contributed, is to be spent. There was consensus that citizens should know the outcomes of decision‐making processes, regardless of level of citizen involvement in the process.

## 4 DISCUSSION

Using a simplified, scenario‐based voting intervention we were able to engage with a large number of citizens on setting priorities for research funding in the field of infection. This method was reproducible and well received during in‐depth analysis by citizens participating in follow‐up focus groups. However, contradictory feelings within our citizen population were also observed. These feelings oscillated between responsibility to do the best for society and what might affect someone locally when allocating resources, and the citizen's view on whether they should be able to participate in deciding on priorities for research based on less information than “experts” have available when allocating importance to research funding applications. The researchers also observed knowledge gaps in terms of geographical proximity for some infections, and lack of understanding that infections can easily become global.

Given the enhanced role of citizens along the involvement continuum, from involvement in delivery of research to providing support on strategic decisions,^2^, ^3^ there is a need to develop and test the feasibility of tools with reproducible methodologies that can be deployed across a number of settings to quickly and accurately map the views of a large number of citizens to inform strategic decision making. The challenge following collection of data similar to that described in this study is how it is then used to influence policy makers and those making strategic decisions for funding. Furthermore, the role of current media attention and parallel awareness campaigns on voting trends must also be considered. Within our study we identified that drug‐resistant infections were a leading priority to the citizens that we surveyed. Whilst our in‐depth analysis work demonstrated that information provided during the voting procedure allowed individuals to weigh options and consider what was important to them when voting, the role of prior knowledge, and that covered in the media was also highlighted as something that could either positively or negatively influence an individuals' decision making. In particular, the role of the media in influencing citizen decision making has been well described in similar fields of infection control and surveillance programmes.^20^, ^24^ Future work must explore how much this influence may play on decision making in the context of our intervention and timing of such interventions.

The discussion regarding relevance of citizens being asked to make such strategic decisions arose at the focus groups but did not surface at the university open day where citizens were asked to cast votes. This difference may be important in informing the appropriateness of voting‐based methods versus group‐based analysis for collecting citizen priorities and preferences for large‐scale strategic decisions. Furthermore, the reported reaction and rejection to being asked to assume responsibility for priority setting reported within our focus groups are a well‐described phenomenon in the literature.^25^ This disparity in opinion has been observed elsewhere during recent large‐scale citizen voting scenarios. For example, the recent UK referendum to leave the European Union, caused much greater division in citizens opinions on the appropriateness of allowing the public to decide the outcome of a large‐scale strategic decision, with concerns over evidence provided to the public to help inform their decision making.^26^ However, positive examples where large‐scale citizen voting has had a positive response is also available through the 2014 Longitude prize, where citizens voted to support drug‐resistant infection research from a shortlist of finalists from a number of different fields after information was provided in an evidence‐based manner on a reputable television show.

Recently, van Bekkum and colleagues described the technocratic approach taken by research bodies in the UK to PPI in decision making.^18^ Here, they describe how funding bodies tend towards selecting citizens' with technical knowledge and expertise in the field that they are involved, which can lead to a narrowing of the focus of PPI in making decisions surrounding funding opportunities. One of the leading reasons for this was due to the practical challenges posed by involving large numbers of citizens in the decision‐making process.^18^ Another was the due to the challenges that members of the public can face when being entered into the scientific environment as a citizen representative.^18^ These were themes common to our reported results, where despite reporting interest in involvement in decision making our own participants also voiced concerns over “non‐experts” ability to make decisions based on what they perceived as limited information. Therefore, as well as providing a new simplified avenue to promote wider citizen engagement in strategic decisions this type of tool may also be positively adapted to demonstrate that broader members of the public can make informed decisions about priorities for research that are congruent with current funding strategies. This can be demonstrated with recent investment in the field of drug‐resistant infections and emerging infections, such as Ebola and Zika virus, which based on the information provided in our scenario citizens also prioritized as important avenues for funding to be allocated currently.

### 4.1 Limitations

There were several limitations to our study. Firstly, the voting was undertaken at a university public festival which may have biased voting given that people attending where likely to have a higher than average educational background. Furthermore, the stand was situated in a building with exhibits on all six infection themes that were included for voting, meaning that citizens may have been influenced by further information provided within the building. Voting was also anonymous meaning that we were unable to collect demographic information on those casting votes. This is something that we aim to address in future evaluation of the tool. Finally, focus group work identified the potential biasing effect of placing the infection themes upon a world map, as individuals may have been more likely to vote for themes with closer proximity to themselves. This may have influenced some of the voting towards drug resistance infections. However, the majority of the six infection themes were placed in geographically relevant locations to where they are currently major issues, meaning that this influence may have been justified if proximity to oneself was a deciding factor for the individual. Further work is now underway to explore alternative methods for displaying this type of information.

Moreover, knowing that 43% of the population are health literate must also be taken into account when refining such tools and the manner in which they are implemented. Improving health literacy remains a priority when thinking about individuals and decisions about their own health, but also as it has implications for the extended roles citizens are being asked to take. Citizens indirectly make decisions which impact on public spending for health and research and overseas development. The tool tested here focused on a specific area of strategic decision making and the level of information that the public were able to assess and assimilate. Our tool development was informed by the health literacy gap. There is further potential for learning by implementing the tool with specific groups with specific/known health literacy needs.

## 5 CONCLUSION

This study demonstrates that this tool provides a useful means of engaging members of the public and also can be used to gauge public confidence in casting votes. The tool may be implemented either in a group setting with discussion, or by allowing space for respondents to state their level of confidence or additional information needs. Voting must be taken in context as a useful addition to the portfolio of current methods for promoting wider citizen engagement in strategic decision making.

## COMPETING INTERESTS

LSPM has consulted for bioMérieux and DNA electronics in 2014. All other authors have no conflict of interests to declare.

## AUTHORS' CONTRIBUTION

All authors contributed significantly towards the planning of this study and production on this manuscript. TMR, RA, EC and ECS ran the scenario and focus groups. All authors were involved in data analysis. TMR drafted the initial manuscript with all authors contributing significantly towards its finalization for submission.

## DATA SHARING

There is no additional, unpublished data available from this study.
