# Supplementary material for: Involving citizens in priority setting for public health research: Implementation in infection research
Source: Health Expect. 2017 Jul 21;21(1):222–9. doi: 10.1111/hex.12604 (PMC5750690; doi:10.1111/hex.12604)
Supplement: Supplementary file 1 [file HEX-21-222-s001.docx]

**Appendix 1**

***Scenario:*** *You have been appointed as the head of an independent global charitable organisation who sponsor research to promote good health for citizens. As part of your first round call for funding you have been allocated £100 million to sponsor research in the field of infection. From hundreds of applications, your organisation has selected six finalists from varying fields of infection research.*

***Task:*** *As head of the organisation it is your job to consider each application and allocate funding to the researchers based on what you feel are priorities for the future.*

***Further information:*** *You can allocated funding to up to two of the finalists (£50 million each) based on what you deem priorities for research in this area.*
