# Supplementary material for: Involving citizens in priority setting for public health research: Implementation in infection research
Source: Health Expect. 2017 Jul 21;21(1):222–9. doi: 10.1111/hex.12604 (PMC5750690; doi:10.1111/hex.12604)
Supplement: Supplementary file 2 [file HEX-21-222-s002.docx]

**Appendix 2**

**Introduction**

*In the world of research a large amount of the funding that we receive is from publically funded sources. However, members of the public, like yourselves, very rarely have the opportunity to have a say as to where and what this money should be spent on.*

*Working with you this evening over the next 30 minutes, we want to try and capture your views on how we spend money and how we should be trying to involve members of the public in deciding where we spend money on research in the future. Tonight we will focus on the field of infectious disease research.*

| Time | Aim | Question plan |
| --- | --- | --- |
| *0-15min* | **Introduction** | ***Show participants the poster that we used. Ask them to consider the 6 topics***   - *Please say your name and briefly tell us about which topic you would select to fund if you had £50 million* ***AND why?*** |
| *20-30min* | **Explore participants views on public involvement in research**  **Determine what level they should be involved**  **Explore ideas, concerns and expectations of public involvement in strategic decisions** | - *Should members of the public have the opportunity to say where money for research is spent?* - *At what point should you be involved?*   *(in the initial application stages (long listing) or once “finalists have been selected” (shortlisting))*   - *Should public have the only say?* - *How much influence should you have on who gets funding?* - *Would you have any concerns about involving the public in the selection process?* - *How do you see this changing how research is funded? Or do you?* - *Do you think that this would change how we go about funding research?* |
| *20-30min* | ***Explore methods for collecting data from large numbers of citizens***  ***Explore views of our current system and how it could be improved***  ***Present findings so far and explore the participants views on whether these seem valid conclusions to draw*** | - *What is your opinion of the poster which we showed you at the beginning of the session for facilitating involvement in strategy decisions?* - *What information should we provide?* - *Was it easy to understand?* - *What other factors influenced your decisions making about what you would fund?*   Show overall results of finding and main conclusions   - *What is your opinion on the findings of our investigation?* - *How do you think this may influence how research funding is allocated in future?* - *Should the public also have the chance to review the overall results and comment on them?* - *Would you trust this method?* - *How else could we present and collect this information?* |
